# Dose adjustment strategy of levetiracetam in pregnant patients with epilepsy: Case report and literature review

**DOI:** 10.1097/MD.0000000000037977

**Published:** 2024-04-26

**Authors:** Renzhu Liu, Lu Xiao, Xiang Liu, Can Xiao

**Affiliations:** a Department of Clinical Pharmacy, Xiangtan Central Hospital, Xiangtan, China; b Department of Children Health Care, The Maternity And Children Health Hospital of Xiangtan City, Xiangtan, China.

**Keywords:** epilepsy, levetiracetam, pregnancy, therapeutic drug monitoring

## Abstract

**Rationale:**

Pregnant patients with epilepsy are prone to preterm delivery, stillbirth, or cesarean section, and their mortality rate is almost 10 times higher than that of normal pregnant women. The potential negative influences of antiepileptic drugs (AEDs) on the fetus are weighed against the necessity for achieving optimal control of seizures. Treatment with AEDs during pregnancy is a major challenge for pregnant women and healthcare teams.

**Patient concerns:**

This paper reports two cases of young women diagnosed with pregnancy and epilepsy.

**Intervention:**

The dose of levetiracetam was adjusted under the guidance of therapeutic drug monitoring to reduce the effects of seizures on the fetus and the incidence of reproductive toxicity caused by adverse drug reactions.

**Outcomes:**

Epilepsy was well controlled in the two pregnant patients, and the newborns had no genetic disorders.

**Lessons:**

It is recommended to regularly monitor the serum LEV level in pregnant patients with epilepsy. This practice serves as a foundation for adjusting the drug treatment plan and offering more precise guidance for medication management during pregnancy.

## 1. Introduction

Epilepsy is a common serious brain disease, influencing more than 700,000 people worldwide.^[1]^ Epilepsy, characterized by abnormal neuronal discharges in the brain resulting from various causes, has an incidence rate of up to 0.7% in pregnant women.^[2]^ Women with epilepsy are at an increased risk of experiencing preterm birth, stillbirth, or undergoing Caesarean section during gestation, with a mortality rate nearly 10 times that of typical pregnancies. Seizures during these epochs can escalate the risk of offspring malformations and neurological disorders.^[3]^ Oral antiepileptic drugs (AEDs) serve as primary epilepsy therapies, necessitating continued therapy for pregnant epilepsy patients, as well as notable teratogenic implications.^[4]^ Fetal AED syndrome manifests across all conventional AEDs, with fetal exposure to AEDs associated with an increased risk of cognitive defects ranging from 1% to 1.4% to 6%.^[5]^ Seizures occurring during pregnancy are detrimental to fetal brain growth. The primary concentration in the medical management of epilepsy during pregnancy is to control and minimize maternal seizures, thereby mitigating potential teratogenic risk to the fetus.

Levetiracetam (LEV), a second-generation broad-spectrum AED, acts by binding to neuronal synaptic vesicle protein 2A. It inhibits calcium exocytosis and opposes negative regulators of gamma-aminobutyric acid and glycine-gated currents, and suppressing excessive neuron synchronization.^[6]^ LEV efficaciously manages epilepsy during pregnancy with no influence on fetal cognitive development. As a widely utilized antiepileptic drug (AED) for women of childbearing age, it is highly recommended for managing epilepsy during pregnancy.

## 2. Case presentation

Case 1: On November 25, 2022, a female patient was admitted to our hospital who had experienced recurrent seizures for 15 years, presenting with sleep seizures and postseizures dizziness and discomfort. The patient experienced a second waking episode characterized by hallucinations (color shadows) after seconds of impaired consciousness, leading to convulsions. She does not have a family history of epileps. Valproic acid was initially prescribed, while in September 2021, it was switched to LEV due to inadequate epilepsy control. The initial dose of LEV at 0.5 g twice daily was subsequently escalated. In September 2022, magnetic resonance imaging of the head showed flat bilateral hippocampi and 24-hour video electroencephalography showed slow waves and sharp slow waves in bilateral frontal, anterior temporal, central and bilateral parietal (front of the head) regions. However, a pregnancy plan necessitated monitoring serum drug levels to precisely control epilepsy and minimize the drug impact on the fetus. On January 25, 2022, serum LEV level was 11.58 mg/L, leading to occasional seizures. The LEV dose was progressively increased to 1 g bid. On November 30, 2022, the serum LEV level was 9.0 mg/L during the first trimester, supplemented with an adequate amount of folic acid at 4 mg. As pregnancy progressed, serum LEV level declined. In April 2023, the measured serum level of LEV was 2.17 mg/L, highlighting a modification of the administration guideline to 1 g earlier and 1.25 g later. The serum LEV level was elevated, which reached 4.79 mg/L in May 2023.

Case 2: A 22-year-old woman began experiencing disturbances in consciousness, limb spasms, and trismus for 15 years before her admission to the hospital, followed by annual episodes of convulsions or handshaking accompanied by unconsciousness. She does not have a family history of epilepsy. These attacks occurred with varying frequency, whereas exhibited responsiveness to calcium and B12 supplements, providing some relief. More than 2 years before becoming pregnant, abnormal EEG findings were improved during and after pregnancy. However, during pregnancy, the patient reported symptoms, such as fatigue, palpitations, cyanosis of the jaw and lips, and brief episodes of consciousness loss. At the time of admission on July 20, 2022, she presented with upper limb tremors, limb muscle aches, and sleep disturbance due to nonuse of AEDs for fear of fetal effects. The patient was hospitalized on August 27, and October 4, as seizures related to pregnancy recurred. During hospitalization, video electroencephalography showed a mildly abnormal EEG, and sporadic sharp slow waves were occasionally seen in the right anterior and middle temporal regions during sleep. Despite hospitalization, she opted against oral AEDs. Subsequently, from October 30 to November 4, she was hospitalized again and received oral LEV for seizure management. The serum drug level was assessed on November 17, leading to a dosage adjustment to 0.75g twice daily due to occasional seizures postmedication. In January 2023, the LEV administration was further adjusted from 1.25 g early and 1 g later to a gradual increase to 1.5 g twice daily. This adjustment was made in response to an elevated serum drug concentration measured at 11.59 and 12.88 mg/L postpartum.

**Figure 1:**
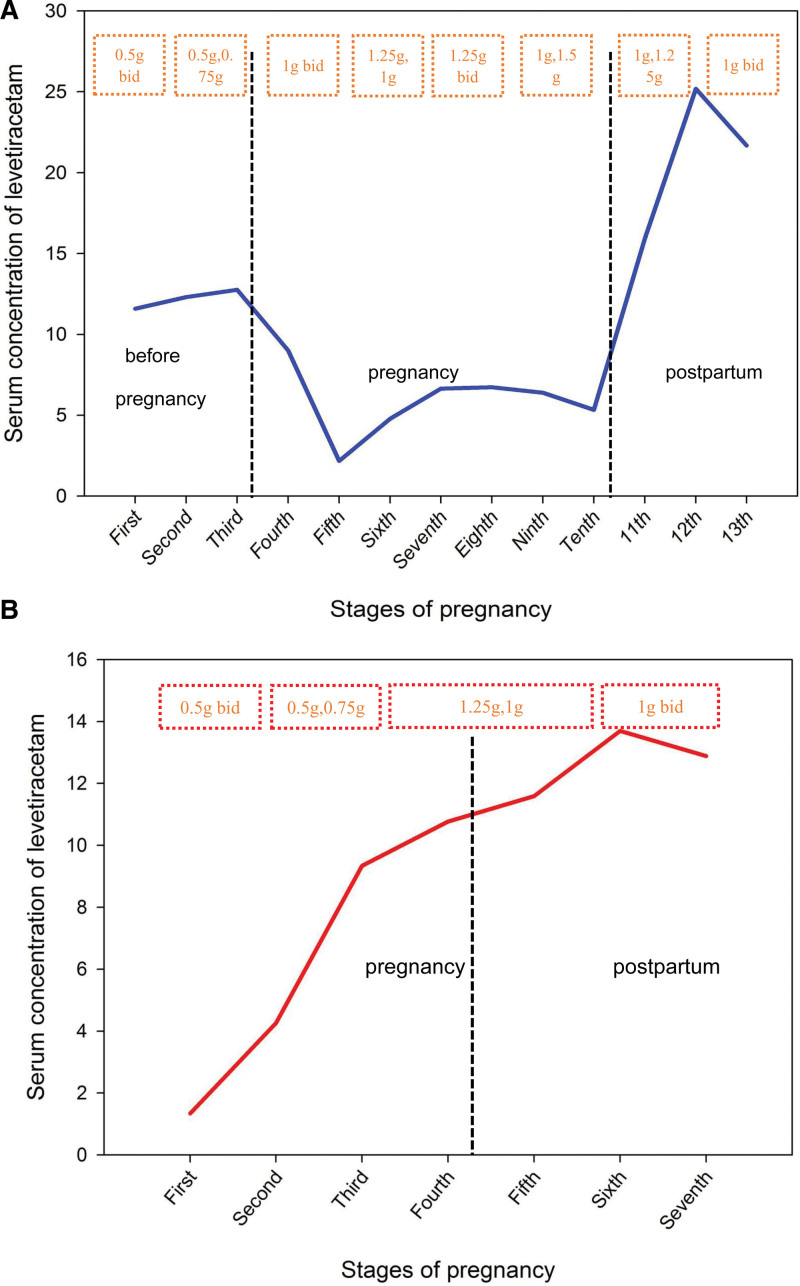
(A) Changes in serum concentration of different doses of levetiracetam in case 1 at different stages of pregnancy. (B) Changes in serum concentration of different doses of levetiracetam in case 2 at different stages of pregnancy.

Normal liver and kidney functions were found throughout pregnancy. Changes in LEV dose and serum drug concentration before, during, and after pregnancy are illustrated in Figure 1.

## 3. Discussion

### 3.1. Comparative study and drug selection regarding the teratogenicity of AEDs during pregnancy

The management of AEDs during gestation poses substantial challenges for both woman and treatment teams. Given the conflicting risks associated with poor seizure control and potential fetal adversities, the management of epilepsy during pregnancy mainly involves a careful consideration of AED teratogenicity and subsequent neurodevelopmental outcomes in the offspring. Table 1 provides an overview of frequently used AEDs with the risk of major congenital malformations.^[7]^

**Table 1:** Frequently used antiepileptic drugs with risk of major congenital malformations.

Antiepileptic drugs	OR (95% CrI)
ETX	3.04 (1.23–7.07)
VPA	2.93 (2.36–3.69)
TPM	1.90 (1.17–2.97)
PB	1.83 (1.35–2.47)
PHT	1.67 (1.30–2.17)
CBZ	1.37 (1.10–1.71)
LTG	0.96 (0.72–1.25)
LEV	0.72 (0.43–1.16)
OXC	1.32 (0.72–2.29)
CLB	3.48 (0.52–13.84)
PRM	1.22 (0.65–2.12)

95% CrI = credible intervals, CBZ = carbamazepine, CLB = clobazam, ETX = ethosuximide, LEV = levetiracetam, LTG = lamotrigine, OR = odds ratio, OXC = oxcarbazepine, PB = phenobarbital, PHT = phenytoin, PRM = primidone, TPM = topiramate, VPA = valproic acid.

### 3.2. Pharmacokinetic changes of AEDs in pregnancy

The general recommendation for the utilization of AEDs in pregnancy is to prescribe the lowest effective dose of AED. In addition, single-drug therapy is superior to multi-drug therapy, and some AEDs recommend the use of therapeutic drug monitoring to guide drug administration.^[8]^ In the present study, in case 1, valproic acid had long been used for antiepilepsy, followed by administration of LEV for its antiepilepsy effect during pregnancy. In case 2, the patient, with a lengthy history of epilepsy, deliberated on the impact of epilepsy drugs on the fetus postpregnancy. Despite irregular adherence to epilepsy medication, experiencing repeated seizures, the patient opted for antiepileptic treatment with LEV under the guidance of a doctor and pharmacist team, emphasizing the importance of medication education. AEDs have specific pharmacokinetic changes during pregnancy. If these alterations are associated with an increased risk of seizures, the dose of AEDs should be adjusted under the guidance of therapeutic drug monitoring.^[9]^ The expansion of plasma volume during pregnancy may result in an increased volume of distribution, potentially leading to a reduction in the concentrations of AEDs. Concurrently, serum albumin level may be decreased due to an increase in plasma volume. Pregnancy-related changes may affect the clearance of AEDs, accompanying by a high protein binding rate.^[10,11]^

### 3.3. Metabolism of LEV in pregnancy

At present, the specific mechanism of the change in clearance rate during pregnancy remains elusive. This mechanism could be attributed to factors, such as frequent vomiting in early pregnancy, which may impact the uptake and intestinal absorption of medications, including LEV. Additionally, the rise in circulating blood volume and the apparent increase in the volume of drug distribution may contribute to a decrease in serum drug concentration. While LEV is not metabolized by the liver in vivo, studies have indicated that enzyme-inducing AEDs can elevate the clearance rate of LEV.^[12]^ Therefore, alterations in liver metabolic enzymatic activity or changes in endocrine status during pregnancy may, to some extent, favor the metabolism of LEV. It is noteworthy that LEV is primarily excreted by the kidneys. During pregnancy, renal blood flow increases by 35% to 65% and glomerular filtration rate is elevated by 50% to 80%, resulting in an increase in LEV clearance rate, while the degree of clearance is worthy of further exploration. Research indicated a noticeable variability in the impact of pregnancy on drugs primarily eliminated by the kidneys, with clearance rates for most drugs showing a range of 20% to 65%.^[13]^

### 3.4. Causes of reduced serum LEV level in pregnancy

In the case of LEV, it is fully absorbed after oral administration, does not undergo metabolism by Cytochrome P450, and it does not bind to plasma proteins. The inactive metabolites produced by nonoxidative metabolism account for 30% of LEV excretion and the remaining 2/3 can be excreted as prototypes in the urinary tract. Due to the decreased gastrointestinal motility and the pregnancy response, absorption is reduced, plasma volume is escalated, protein binding capacity is reduced, drug distribution is elevated, and serum drug concentration is reduced. The escalation of liver metabolism, elevated renal blood flow, and an increased glomerular filtration rate may contribute to augmented renal excretion and other factors leading to a reduction in LEV concentration during pregnancy.^[14]^ In case 1, the mean dose of LEV was 0.625 g bid and the mean serum drug concentration was 12.21 mg/dL. The mean dose of LEV during pregnancy was 1.25 g bid and the mean serum drug concentration was 5.27 mg/dl. Due to the influence of pregnancy, the apparent volume of drug distribution also rises, resulting in increased drug clearance. At the same dose of LEV, the serum drug concentration was reduced by 363%. The mean postpartum dose of LEV was 1.0 g bid, and the mean serum drug concentration was 20.93 mg/L. The postpartum serum drug concentration and the prepregnancy serum drug concentration were basically the same at the same dose. In case 2, it was infeasible to accurately determine the prepregnancy baseline level of LEV because the patient did not take LEV in the early stages of pregnancy, while the postpartum serum drug concentration of LEV was significantly higher than that during pregnancy, which was basically similar to case 1.

### 3.5. Strategies for dose adjustment of LEV in pregnancy

During pregnancy, it is mainly necessary to adjust the dose of LEV to maintain the target concentration. In cases 1 and 2, the dose was significantly escalated in the middle and late stages of pregnancy to prevent a rapid drop in serum drug concentration, leading to seizures. postdelivery, both LEV clearance and apparent volume of distribution promptly revert to prepregnancy levels. It is essential to monitor the serum drug concentration to evaluate the dose of administration to avoid drug toxicity due to the increased dose during pregnancy. In clinical practice, prior to conception, baseline concentrations of AEDs, including LEV, are measured twice, and a 15% to 25% reduction from the prepregnancy baseline may not warrant dose adjustments during pregnancy. If the AED concentration changes by more than 25% compared with the baseline concentration, dose adjustment is recommended.^[15]^ Concurrently, postpartum serum drug concentration should be monitored. If the same dose is significantly higher than the prepregnancy dose, the dose should be gradually adjusted to prevent the rapid decrease in serum drug concentration causing seizures. This study is a retrospective analysis on cases. Due to clinical unpredictability, LEV serum levels were not baseline-tested in case 2 before conception. Future investigations will gather additional cases to examine LEV clearance throughout pregnancy for accurate epilepsy control during all stages.

## 4. Conclusions

Significant changes in the pharmacokinetics of LEV could occur during pregnancy, marked by a substantial increase in clearance rate and apparent volume of distribution. These parameters gradually returned to prepregnancy levels after delivery. Regular monitoring of serum drug concentrations, particularly for LEV, is recommended for pregnant epilepsy patients. This monitoring provides a foundation for adjusting treatment plans, ensuring more accurate guidance for medication management during pregnancy for patients with epilepsy.

## Author contributions

**Conceptualization:** Xiang Liu, Can Xiao.

**Data curation:** Renzhu Liu, Lu Xiao.

**Funding acquisition:** Xiang Liu.

**Methodology:** Lu Xiao.

**Writing – original draft:** Renzhu Liu.

**Writing – review & editing:** Xiang Liu, Can Xiao.
